# Aerosol demasking enhances climate warming over South Asia

**DOI:** 10.1038/s41612-023-00367-6

**Published:** 2023-05-20

**Authors:** H. R. C. R. Nair, Krishnakant Budhavant, M. R. Manoj, August Andersson, S. K. Satheesh, V. Ramanathan, Örjan Gustafsson

**Affiliations:** 1 Department of Environmental Science and the Bolin Centre for Climate Research, Stockholm University, Stockholm, Sweden; 2 Maldives Climate Observatory at Hanimaadhoo, H. Dh. Hanimaadhoo, Maldives; 3 Divecha Centre for Climate Change, Indian Institute of Science, Bangalore, India; 4 Centre for Atmospheric and Oceanic Sciences, Indian Institute of Science, Bangalore, India; 5 DST-Centre of Excellence in Climate Change, Indian Institute of Science, Bangalore, India; 6 Scripps Institution of Oceanography, University of California San Diego, La Jolla, CA USA

**Keywords:** Atmospheric chemistry, Climate-change impacts

## Abstract

Anthropogenic aerosols mask the climate warming caused by greenhouse gases (GHGs). In the absence of observational constraints, large uncertainties plague the estimates of this masking effect. Here we used the abrupt reduction in anthropogenic emissions observed during the COVID-19 societal slow-down to characterize the aerosol masking effect over South Asia. During this period, the aerosol loading decreased substantially and our observations reveal that the magnitude of this aerosol demasking corresponds to nearly three-fourths of the CO_2_-induced radiative forcing over South Asia. Concurrent measurements over the northern Indian Ocean unveiled a ~7% increase in the earth’s surface-reaching solar radiation (surface brightening). Aerosol-induced atmospheric solar heating decreased by ~0.4 K d^−1^. Our results reveal that under clear sky conditions, anthropogenic emissions over South Asia lead to nearly 1.4 W m^−2^ heating at the top of the atmosphere during the period March–May. A complete phase-out of today’s fossil fuel combustion to zero-emission renewables would result in rapid aerosol demasking, while the GHGs linger on.

## Introduction

Anthropogenic climate change is an acute global challenge, demanding international attention and cooperative solutions^1^. The main culprit is anthropogenic emissions of greenhouse gases (GHG) such as CO_2_. In the past four decades, the atmospheric loading of CO_2_ has increased by 50%, causing a 1 K increase in global temperatures^2^. In response, worldwide efforts are now considered and enacted to reduce emissions, including the Paris agreement^3^. While the warming impact of GHGs is well understood, the climate effects of aerosols are less well characterized, contributing to large uncertainties in e.g., radiative forcing^4^. Overall, aerosols cool the climate, either directly through interactions with solar radiation or by aerosol-cloud interactions^5–7^. The extinction of shortwave radiation by atmospheric aerosols reduces the surface-reaching solar radiation. The magnitude of this extinction is directly proportional to the columnar loading of aerosols and in turn, leads to a reduction in the longwave radiation emitted by the earth’s surface^8^. This results in the masking of global warming (reduction of total warming), thus contributing to net climate cooling. The magnitude of this aerosol-induced cooling effect remains highly uncertain due to the complexity of the composition and lifecycles of aerosols^8^.

Climate change mitigation policies, naturally targeting emissions of longer-lived greenhouse gases (CO_2_, CH_4_ and N_2_O), will also decrease co-emitted “climate cooling” aerosols, which are much more shorter-lived and thus will decrease more quickly^9^. This consequence of mitigation—demasking the aerosol cooling, will lead to net climate warming and thus inadvertently countering the intended impact—has so far received little attention in the climate policy arena. Such warming and changes in the earth’s radiative budget would have many severe effects, including disturbance to global biogeochemical cycles. In this context, the observational quantification of aerosol demasking is equally desired by both policymakers and scientists.

Based on the collective understanding of the relative role of GHGs and aerosols on the radiative forcing of climate change, the IPCC report^1^ summarizes that the change in the radiative forcing from pre-industrial period to the present following the increase of all greenhouse gases is +4.1 W m^−2^ (±0.8 W m^−2^) with CO_2_ forcing contributing 2.1 W m^−2^ and non-CO_2_ forcing 2.0 W m^−2^.The estimated forcing due to the increase in aerosols is −1.4 W m^−2^ and spans a much wider range in uncertainty (0 to −2.8 W m^−2^)^1,2^. Direct observational data on this aerosol masking effect is lacking. The inadvertent demasking experiment caused by the COVID lockdown in South Asia (SA) provided an opportunity to constrain this process.

The 2019 outbreak of the coronavirus disease (COVID-19) and its rapid spread worldwide made the World Health Organization (WHO) declare it a pandemic on 11 March 2020^10^. Stringent restrictions were introduced globally, with most affected countries enforcing lockdowns and behavioral restrictions, e.g., confining the public to their homes. As a result, the levels of multiple archetypical short-lived anthropogenic air pollutants, e.g., NO_X_ (NO + NO_2_) and PM_2.5_ (fine particulate matter), plummeted in the affected regions^10–16^. Meanwhile, the levels of long-lived GHG remained much less perturbed. This constitutes a unique opportunity—a large-scale “geophysical experiment”—to empirically investigate and constrain the impacts of anthropogenic short-lived aerosols on the regional climate. Specifically, we focus on the high pollution regime of SA, where large effects of aerosol-induced anthropogenic climate masking effects are expected.

**Fig. 1:**
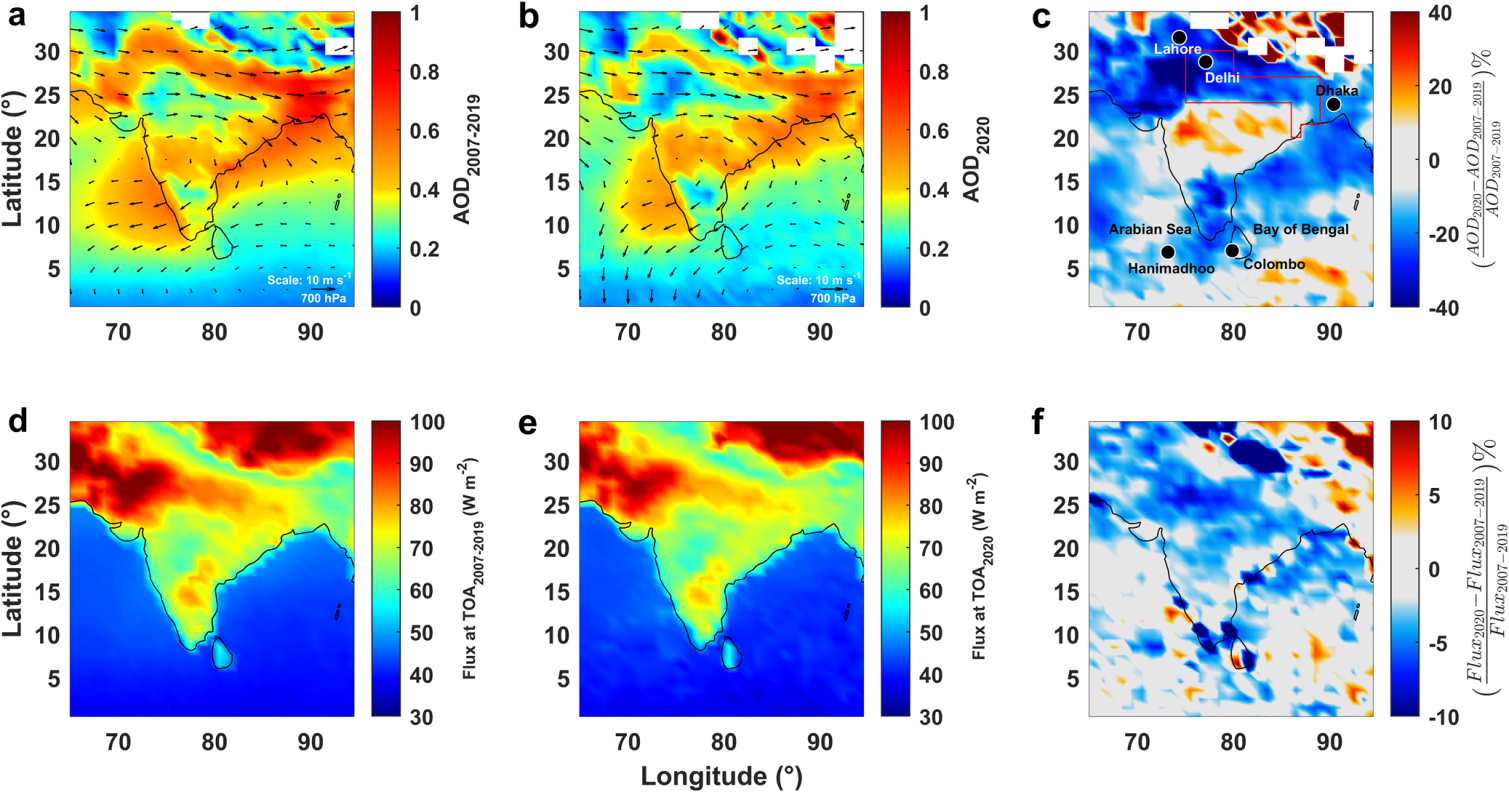
Reduction in aerosol loading and top of the atmosphere flux. Measurements during the period 24 March to 31 May. **a** Mean Aerosol Optical Depth (AOD) for the period in the years 2007 to 2019. **b** Mean AOD for the period in 2020. **c** Mean relative change of AOD_550 nm_ retrieved using MODerate Resolution Imaging Spectroradiometer (MODIS) instrument aboard the Aqua satellite for the period. The black arrows embedded in panels **a** and **b** represent mean wind vectors for the corresponding period at 700 hPa. The box (red color) in panel **c** indicates the region of Indo-Gangetic Plain. **d** Mean Top of the Atmosphere (TOA) clear sky flux for the periods in the years 2007 to 2019. **e** Mean TOA clear sky flux for the period in 2020. **f** Mean Relative change of TOA clear sky flux for the period retrieved using CERES onboard Aqua and Terra satellites.

This study probes the aerosol masking effect by investigating aerosol loadings and the radiation balance in the entire South Asian region during the COVID-19 slow-down period, compared to previous years. The region captured in our study is South Asia (0–35°N and 60–95°E), which includes India, Pakistan, and Bangladesh. In addition, we detail the changes over the Indo-Gangetic Plains (IGP; 20–30°N and 75–84°E), an extensive region that includes eastern Pakistan, the entire north Indian region, and western Bangladesh, and with a population of over 650 million. The IGP is the most polluted large-scale region of SA (and perhaps the world). Due to the general shortage of adequate ground-based data, we rely on satellite data for the columnar loading of aerosols and top-of-the-atmosphere radiative forcing. This is complemented by continuous data from the Maldives Climate Observatory Hanimaadhoo (MCOH); a receptor site for the outflow of aerosols and other air pollution from South Asia^17,18^. Satellite observations were compared with ground measurements at MCOH and were found to be in good agreement. Confidence in the inferences from satellite observations rests on this inter-comparison analysis. During the March to May period considered in this study, air masses at MCOH originated 89% of the time from Pakistan, IGP, and SW coastal regions of India (Supplementary Figure 1) and included air masses from mega-cities such as Mumbai, Kolkata, and Dhaka (Fig. 1a–c). The observations include aerosol number density, black carbon mass concentration, radiation fluxes, scattering coefficient, absorption coefficient, and the chemical composition of aerosols. Additionally, a radiative forcing model^19^ was used, which was constrained with the ground and satellite observations, to estimate the aerosol radiative forcing. Hence our findings of demasking of the aerosol forcing are mostly based on direct observations, yet we rely on the combination of MCOH data and model estimated forcing to interpret the factors contributing to the observed changes.

## Results

### Reduced aerosol loading in South Asia

The impact on vertically-integrated aerosol number concentrations is reflected in the aerosol optical depth (AOD)^20^. AOD measured by satellites is the only globally-observed metric of aerosols and is the vertically integrated product of aerosol number concentrations and the extinction coefficient of the aerosols. During March-April-May (MAM) 2020, the regional loadings of aerosol optical depth (AOD) over SA were significantly lowered (up to ~40%) (Fig. 1a–c). The AOD values over SA during MAM 2020 decreased by 19% (0.08 ± 0.1), 10% (0.04 ± 0.1), and 8% (0.03 ± 0.1), respectively, compared to the corresponding period of the years 2018–2019, 2007–2019 and 2003–2019 (Table 1, Supplementary Tables 1–2). The relative shift is largest in regions where the anthropogenic contributions to high AOD are substantial, such as in the IGP (Fig. 1c). The AOD over the highly polluted IGP decreased by 18% (0.1 ± 0.1), 10% (0.05 ± 0.1) and 8% (0.04 ± 0.1) during MAM 2020 compared to previous sets of years 2018–2019, 2007–2019, and 2003–2019, respectively (Table 1, Supplementary Tables 1–2). Vectors representing the synoptic wind (700 h Pa) over SA during MAM 2007 to 2019 and MAM 2020 embedded in Fig. 1a, b reveal only weak changes with magnitudes similar to the interannual variability. The temperature variations and relative humidity measurements at different ground stations over SA during MAM 2020 similarly showed only trivial variations compared to the same period during previous years^21,22^ and are in good agreement with the satellite observations (detailed in Supplementary Note 1). Taken together, these results confirm a substantial reduction in the columnar aerosol load over SA due to the reduced economic and transport activities during the pandemic slow-down^21–25^.

**Table 1:** The changes in aerosol optical depth, surface- and top of the atmosphere-forcing.

Region	Change in aerosol optical depth (unitless)	Surface brightening (change in surface forcing; W m^−2^)	Demasking (change in top of atmosphere forcing; W m^−2^)
Entire South Asia	−0.07	+12^a^	+1.4
Indo-Gangetic Plains	−0.1	+16^a^	+2.3
Maldives Climate Observatory (MCOH)	−0.14	+18	+1.0

Values were estimated by the difference between 24 March to 31 May 2020 versus the average of the corresponding periods of 2018 and 2019. Statistical significance and comparison with longer multi-year time series for several parameters (all shown in Supplementary Tables 1–5) support these findings.

^a^Estimated with a radiative transfer model using satellite data as input. All other entries in the table are observed values.

**Fig. 2:**
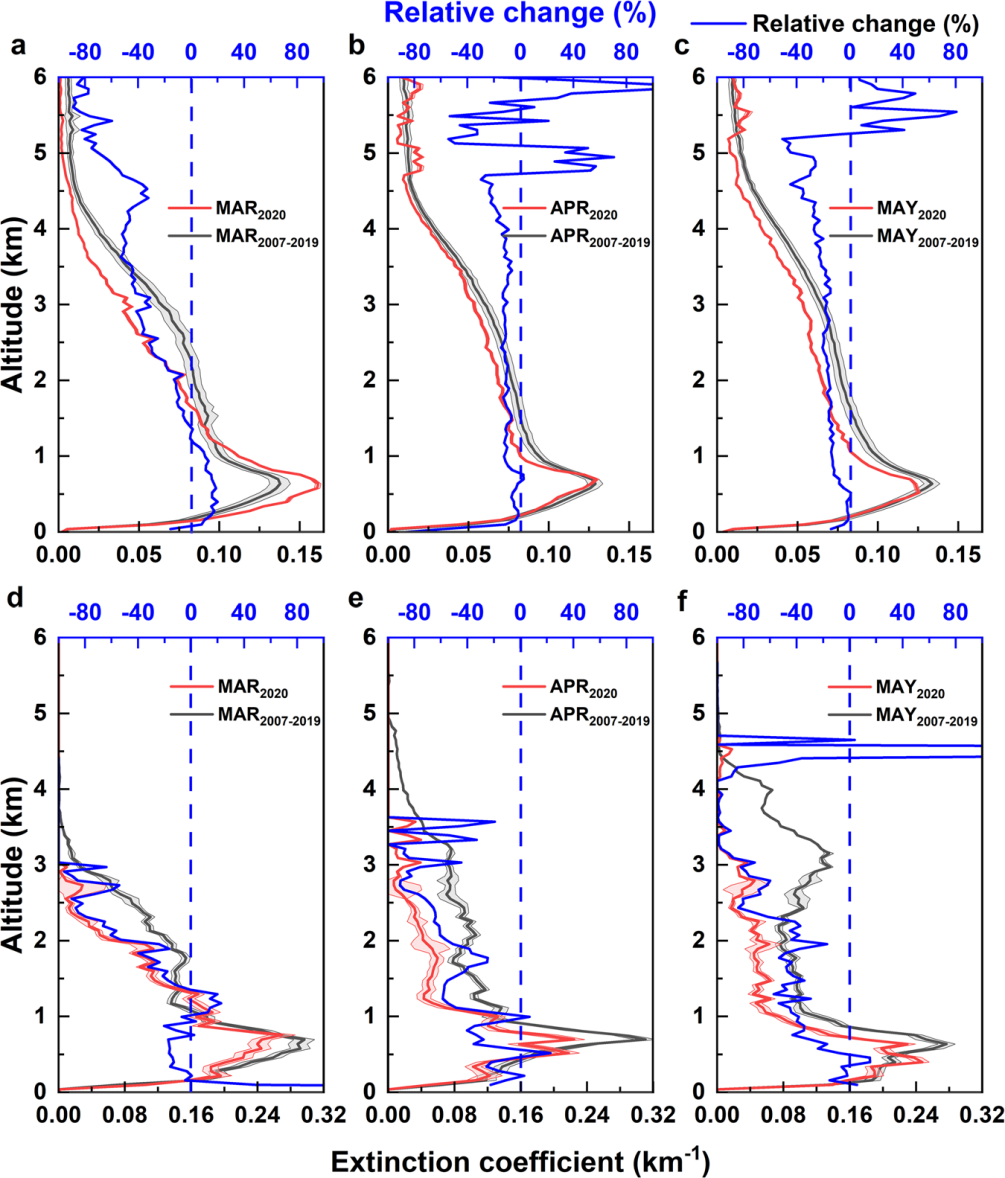
Vertical profiles of aerosols over South Asia (SA) and Maldives Climate Observatory (MCOH). Aerosol extinction coefficient profiles during the period 24 March to 31 May at 550 nm retrieved using Cloud-Aerosol Lidar with Orthogonal Polarization (CALIOP) onboard CALIPSO and weighted by the AOD for the corresponding period for the months March, April, and May over SA and MCOH are shown in panels (**a**–**f**), respectively. Panels **a**–**c** represent profiles over SA and panels **d**–**f** represent profiles over MCOH. The solid red lines and black lines represent the extinction coefficient profiles during the periods 2020 and 2007–2019, respectively. The shaded portion represents the standard error of the measurements. The solid blue lines in each panel show the relative change in extinction coefficient.

Concurrent changes in the vertical distribution of aerosols are often observed with changes in AOD. Several studies have reported the presence of elevated aerosol layers over SA and its role in altering the climate system^7,26–29^. The vertical distribution of aerosols was investigated using altitude-resolved aerosol concentration measurements from Cloud-Aerosol Lidar with Orthogonal Polarization (CALIOP) onboard Cloud-Aerosol Lidar and Infrared Pathfinder Satellite Observations (CALIPSO) satellite. Compared to the lower altitudes, the vertical profile of aerosol extinction coefficient shows a statistically significant decrease at altitudes ranging from 1 to 5 km (Fig. 2a–c) and is consistent with the decrease in MODIS AOD observations. The mean aerosol extinction coefficients in this altitude range over SA during MAM 2020 decreased by ~20% compared to MAM 2007–2019. This reduction in the aerosol loading due to COVID-19-induced societal slow-down can influence the outflow from SA which occurs at altitudes above 1 km (Fig. 2a–c).

**Fig. 3:**
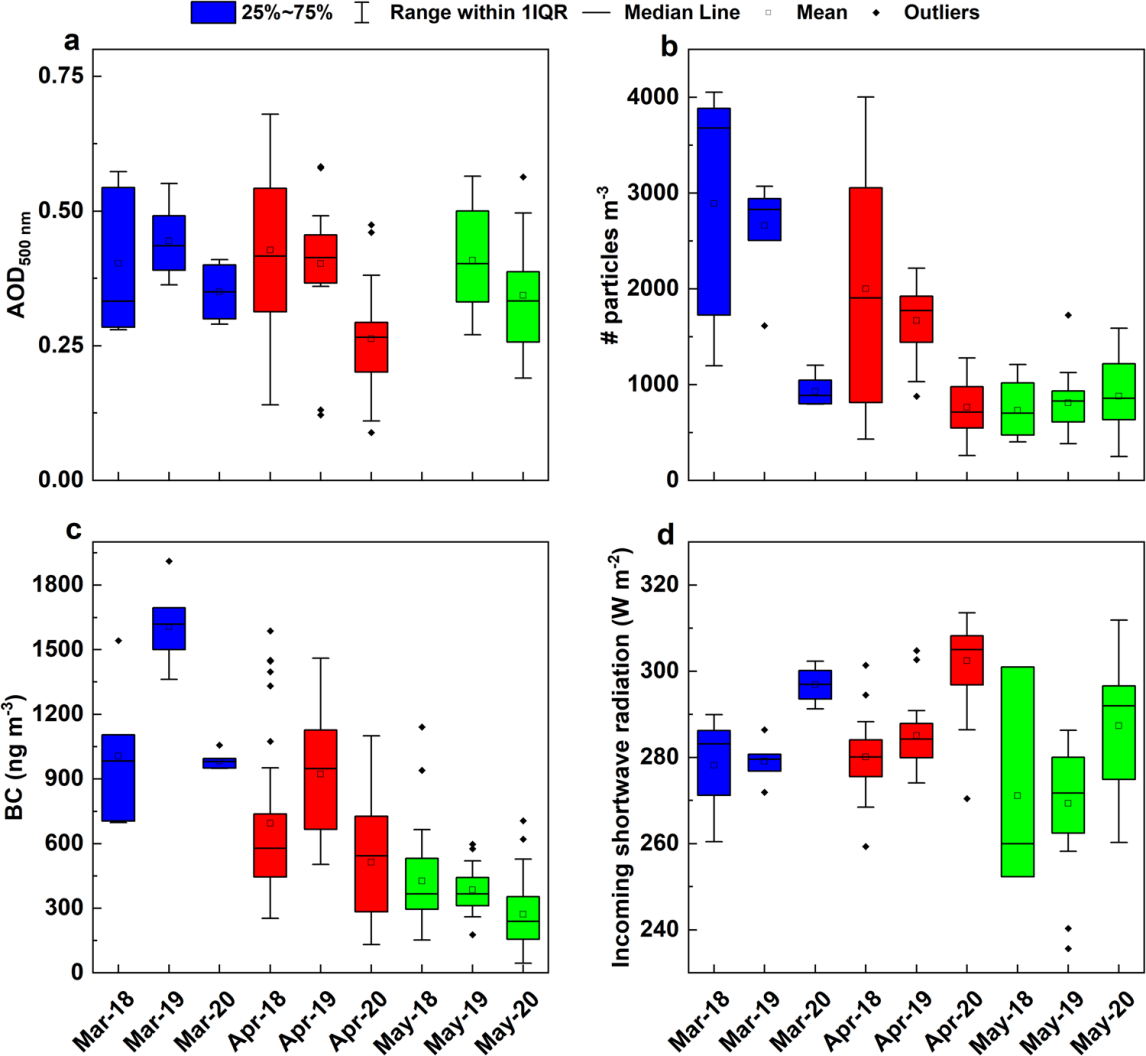
Changes in surface and columnar aerosol properties. Observations over Maldives Climate Observatory Hanimaadhoo (MCOH). **a** Aerosol Optical Depth ground measurements obtained from CIMEL sun photometer (AERONET network). **b** Particle number concentration (cm^−3^) measured using Condensation Particle Counter. **c** Black carbon measurements performed using Aethalometer (880 nm). **d** The incoming short-wave solar radiation measured using CMP21 pyranometer. The boxes and whiskers represent the 25–75 percentile and inter-quartile range, respectively. The mean and median are represented by the black square and line, respectively, while the red cross represents the outliers.

The relative decrease in AOD observed over SA (Fig. 1c) was further investigated using ground-based measurements from MCOH, a regional receptor site capturing the outflow from the IGP during the study period^17,30^ (Fig. 1a, b). The MODIS AOD observations during MAM 2020 revealed a decrease of ~14% (0.05 ± 0.03) over MCOH compared to the past two years (MAM 2018 and 2019) with the corresponding decrease in the actual ground-based AERONET AOD was 33% (0.14 ± 0.13) (Fig. 3a, Supplementary Table 3). In general, MODIS retrievals underestimate the AOD compared to ground-based measurements^31^ possibly underestimating the masking effect of aerosols.

Other ground-based measurements of aerosol parameters at MCOH also reflected signatures of decreased aerosol emissions and atmospheric loadings due to the societal slow-down. Aerosol number and black carbon (BC) concentrations at MCOH decreased by ~48% (758 ± 515 cm^−3^) and ~60% (690 ± 490 ng m^−3^), respectively, during MAM 2020 compared to the preceding two years (Fig. 3b, c, Supplementary Table 3). Along with the decrease in the concentration of aerosols, their optical properties also changed, leading to a significant increase in the single scattering albedo (SSA) by ~10% (0.09 ± 0.05) during MAM 2020, indicating a decline in the transport of anthropogenic BC reaching MCOH (Supplementary Table 3). This change in SSA directly observed from the ground-based measurements at MCOH were not discernable from satellite observations. The SSA (500 nm) indirectly derived using Ozone Monitoring Instrument (OMI) onboard Aura satellite returned negligible changes over SA, which was inconsistent with the more direct measurements at MCOH. This discrepancy can be attributed to the increasing uncertainty in the indirect OMI estimates of SSA, especially under decreasing AOD and its low sensitivity of detection at the lower altitudes^32,33^. The vertical distribution of aerosols over MCOH retrieved using CALIPSO satellite shows significant decrease in the aerosol extinction above 1 km altitude (Fig. 2d–f). The contribution to the AOD from this altitude range changes from 42.5% to 24.8% during the slow-down period. A summary of satellite observations and statistics during the period of study and its comparison over IGP and SA is provided in Supplementary Tables 1–2. We performed t-test to evaluate the statistical significance of these changes in AOD. The changes observed in AOD over the entire SA, IGP, and MCOH were statistically significant with *p* (t) values 2.3 × 10^–10^ (7.1), 4.9 × 10^–4^ (3.6), and 0.007 (2.8), respectively, compared to the 2007–2019 period.

### Surface brightening and radiative forcing

During the pandemic societal slowdown period, Clouds and the Earth’s Radiant Energy System (CERES) observations unveiled a substantial decrease in the Top-of-Atmosphere (TOA) fluxes over the whole SA and over IGP (Fig. 1d–f). The changes in the clear sky SW outgoing TOA fluxes during the slow-down and the corresponding periods of 2018–2019, 2007–2019, and 2003–2019 are shown in Supplementary Tables 1–2. A major part of the South Asian landmass and the oceanic region experienced a ~5% decrease in the TOA fluxes during MAM 2020 (Fig. 1f). This decrease was close to ~10% in the IGP and its outflow regions (the northern Bay of Bengal, northern Indian Ocean, and MCOH). The decrease in the AOD (Fig. 1a–c) reduced the TOA fluxes and led to an increase in the surface reaching solar radiation (Supplementary Figure 2) i.e., surface brightening. The surface albedo and clear sky albedo shows no significant changes during MAM 2020 compared to the same period in preceding years (Supplementary Note 2). Hence the observed significant changes in clear-sky TOA fluxes are not confounded by the anomalies of meteorology and albedo variations (Supplementary Note 1 and 2). We performed t-test to evaluate the statistical significance of these changes in TOA fluxes (Supplementary Figure 3).

Strong signatures of these changes are also observed in the surface measurements at MCOH. The surface-reaching solar radiation in the wavelength range 0.28–2.8 μm measured using a pyranometer increased by an average of ~7% (20 ± 12.6 W m^−2^) relative to 2018–2019 (Fig. 3d; Supplementary Table 3). A large reduction in ground-observed Aeronet AOD (~33%) at MCOH reflects lowered light extinction in the atmosphere, thereby increasing the solar intensity at the surface: this is direct observational evidence for the aerosol masking effect (Fig. 3d, Supplementary Figure 2).

The shortwave incoming flux measurements from pyranometer observations at MCOH and the outgoing TOA flux retrieved using the CERES satellite were used to calculate surface forcing and TOA forcing following common approaches detailed elsewhere^34^. We estimated a decrease of ~48% (18.1 ± 17.5 W m^−2^) in surface forcing and a decrease of ~9% (1 ± 1.8 W m^−2^) in TOA forcing (Table 1, Supplementary Table 3). To further investigate the aerosol demasking climate effects over SA and IGP, we also combined our results with Santa Barbara DISORT Atmospheric Radiative Transfer (SBDART)^19^ model using a hybrid approach combining ground and satellite observations (detailed in Methods). The model was validated for MCOH using the pyranometer and CERES observations (Supplementary Table 4). The monthly mean variations of aerosol radiative forcing (MAM, 2018 to 2020) from observations and model are shown in Supplementary Figure 4a and b, respectively. AOD observations were unavailable for the month of May 2018 due to instrument issues. The model revealed significant decreases in forcing of ~13% (1.7 ± 4.8 W m^−2^) at the TOA and ~39% (14.1 ± 10.1 W m^−2^) at the surface, respectively, indicating a shift in the regional radiative balance (Supplementary Table 5).

The corresponding estimates of the atmospheric heating rate, forcing ratio (ratio of surface to TOA forcing (DRF_SF_/DRF_TOA_)) and forcing efficiency (radiative forcing per unit AOD) (MAM, 2018 to 2020) are shown in Supplementary Figure 5 (Supplementary Table 5). The forcing ratio, which was 2.7 ± 0.5 during 2018 and 2019 (similar to earlier measurements in the region)^34^, was reduced to 1.9 ± 0.6 during 2020, concomitant with a reduction in the atmospheric forcing efficiency (~39 ± 38%). The corresponding decrease in the atmospheric columnar heating rate at MCOH during MAM 2020 was ~55% (0.4 ± 0.3 K d^−1^) (Supplementary Table 5). The TOA forcing estimates retrieved using CERES unveiled a substantial decrease of ~16% (2.3 ± 2.1 W m^−2^) and ~13% (1.4 ± 0.9 W m^−2^) over IGP and SA, respectively (Supplementary Table 1). Assuming that the vertical variations in the aerosol composition over MCOH is similar to that over IGP and SA, the model was extended (after constraining the TOA fluxes using CERES observations) to investigate the changes in the radiative balance. Model estimates over IGP revealed a reduction of ~34% (15.8 ± 2.2 W m^−2^) and ~14% (~2.1 ± 5.1 W m^−2^) in the surface forcing and TOA forcing, respectively (Supplementary Table 5). Over SA, decreases of 34% (11.7 ± 7 W m-2) and 12% (1.4 ± 3.2 W m^−2^) were obtained using the model in the surface and TOA forcing, respectively, reducing the columnar heating rate by 0.4 ± 0.2 K d^−1^ (~46%; Supplementary Table 5). The model estimates over SA and the regional variations over MCOH and IGP indicate spatial heterogeneity in the changes during the slow-down period. The statistics of the model estimates of different aerosol radiative forcing parameters and its variations over IGP, SA, and MCOH are provided in Supplementary Table 5.

## Discussion

### Scientific and policy implications

The findings in this study have significant implications for both our scientific understanding as well as for climate mitigation policy. Earlier studies have demonstrated COVID-19 shutdown induced changes in ambient air quality^35^, primarily due to a reduction in the consumption of fossil fuel in the transportation sector^23,36^. The lifetime of the majority of tropospheric aerosols is typically around a week and as a result, the aerosol loading across SA (from surface to at least 8 km in altitude) decreased by as much as 18% (Table 1) following the COVID lockdown. The comparable decrease in atmospheric CO_2_ during 2020 is about 1%^37^. This is the fundamental reason for the here detailed aerosol demasking effect on climate. Mitigation strategies focusing on the phase-out of fossil fuels will lead to quick removal of the short-lived aerosols while the longer-lived major greenhouse gases decrease much more slowly, likely resulting in undesired net warming of the climate during a decades-long transition period. This transitory dilemma has so far received little attention yet ought to be recognized in the climate policy arena.

The second implication concerns the science of aerosol radiative forcing. The 18% decrease in the columnar aerosol loading, revealed by the large-scale geophysical perturbation experiment resulting from the COVID-19 shutdown, led to an increase in radiative forcing by 1.4 W m^−2^ when averaged over SA for the springtime (Table 1). This is about three-fourths of the CO_2_ induced radiative forcing of 1.8 W m^−2 2^. If this were to happen over wide scales, as we would expect from a 100% switchover from fossil fuels to zero-emission renewables, the net radiative heating would increase drastically. This estimate also provides an opportunity for testing IPCC model predictions against observation. The observations broadly support the IPCC model predictions that aerosols have a net cooling effect on climate, with the implication that reducing aerosol sources would lead to net warming^38^, as here quantified by observations. The major surprise from the study is the magnitude of the COVID shutdown-induced increase in surface-reaching solar radiation, the surface brightening, of the order of 15–20 W m^−2^. This surface brightening has major implications for the regional climate, especially the monsoonal circulation^39^, atmospheric circulation^24,40^, and precipitation over SA, and likely also for East Asia and all tropical regions. Other recent studies^41^ also reported weakening of the aerosol cooling effect due to the Covid-19 lockdown and a subsequent short-term warming effect. Despite a strong increase in the observed surface forcing, corresponding changes in the near-surface temperature over the land areas of SA were not resolved from satellite observations (Supplementary Figure 6a). However, sea surface temperatures increased partially in the Bay of Bengal region^42^.

In summary, demasking the aerosol-induced surface cooling through climate mitigation actions will unveil the actual magnitude and effect of GHG-induced global warming; we shall anticipate a decades-long transitory increase in surface temperatures from planned mitigations. The global scale reductions^16^ in the aerosol loading during COVID shut-down provided this unique opportunity to witness and gauge this inadvertent impact of climate mitigation strategies.

## Methods

### Regional outflow

The Maldives Climate Observatory at Hanimaadhoo (MCOH) situated remotely at the northern tip of the island of Hanimaadhoo, Maldives (Fig. 1c) in the Indian Ocean, captures the pollution outflow from the South Asian region^17,18^. The synoptic wind during March-May, 2007 to 2019 and March-May 2020 for SA are shown in Fig. 1a and b, respectively. The polluted airmass from IGP is advected to MCOH during the period March-May (Fig. 1a, b). The general meteorology of MCOH during the study period is provided in Supplementary Table 6, Supplementary Figure 7. Details of the back-trajectory analysis over MCOH during the period of study are shown in Supplementary Figure 1. More details of the long-range transport of pollutants to MCOH and its seasonality is available elsewhere^43,44^. In this study, the ground observations during the societal slow-down period (March 24–May 30, 2020) were compared with the mean of the same period of the previous two years (2018 and 2019) while the satellite observations were compared with the mean of more than a decade (2003 to 2019).

### In situ measurements and satellite remote-sensing

The Covid-19 societal slow-down caused synoptic-scale changes in the emission over SA. The ground-based measurements used in this study were obtained from MCOH. AOD measurements from the CIMEL sunphotometer (Level-2, AERONET network), incoming surface-reaching solar radiation measurements in the wavelength range 0.28 to 2.8 µm using pyranometer (CMP21, Kipp, and Zonen), black carbon (BC_880 nm_) measurements using aethalometer (AE33, Magee Scientific), scattering coefficient at 525 nm from nephelometer (Ecotech, Model M9003), particle number concentration using a condensation particle counter (TSI, Model 3786), and the in situ observations from the weather station were processed, analyzed and used in this study following methods elsewhere^26,34,45–47^. Data from CIMEL sunphotometer was not available during May 2018. Incoming shortwave radiation data from the pyranometer was cloud corrected following the threshold method as detailed in Supplementary Note 3.

We used satellite remote-sensing measurements to delineate the impact of pandemic societal slowdown over SA. The level-3 data products from MODerate Resolution Imaging Spectroradiometer (MODIS) instrument aboard the Aqua satellite was used to retrieve the AOD at 550 nm (MYD08_M3, 1° × 1° resolution, Version-6.1)^48^, surface reflectance (MODISA_L3m_RRS_2018_Rrs, 4 km resolution, L3mVersion-2018)^49^ for wavelengths 412, 443, 469, 488, 531, 547, 555, 645, 667 and 678 nm. The vertical profile of aerosol extinction coefficient was obtained from the measurements of Cloud-Aerosol Lidar with Orthogonal Polarization (CALIOP) onboard Calipso satellite (Level-2, Version-4.2, a horizontal resolution of 333 m and a vertical resolution of 30 m in the troposphere)^20^. The surface-reaching short-wave radiation was obtained using Modern-Era Retrospective analysis for Research and Applications (MERRA) reanalysis data (MERRA-2 tavgM_2d_rad_Nx: 2d, Monthly mean, Time-Averaged, Single-Level, Assimilation, Radiation Diagnostics V5.12.4 (M2TMNXRAD))^50^. The outgoing flux at the TOA was obtained from Clouds and the Earth’s Radiant Energy System (CERES) instruments on board the Terra and Aqua satellites (Terra+Aqua Edition 4.1 SYN1deg dataset, 1° × 1° resolution, Ed4A version)^51^. The aerosol single scattering albedo at 500 nm were retrieved using Ozone Monitoring Instrument (OMI) on board Aura satellite (OMAERUVd_003, 1° × 1° resolution, version e003)^52^. Over SA and the Indian subcontinent, the uncertainty in OMI retrieved SSA can be ±0.05^52^. Under unit AOD conditions this translates to ±9% and ±11% uncertainty, respectively, in the surface and top of the atmosphere forcing estimates over SA. The synoptic wind data was obtained from NCEP_Reanalysis-2 data provided by the NOAA/OAR/ESRL PSL, Boulder, Colorado, USA.

### Impacts of clouds and natural aerosol variability

Clouds and natural aerosol variability affect the quantification of solar dimming. We used clear sky observations to avoid the impact of clouds. Pyranometer observations at MCOH were cloud screened as detailed in Supplementary Note 3. We had sufficient cloud-free pyranometer data samples for significance study (n_MCOH_ = 34 for 2020 and n_MCOH_ = 71 for 2018 & 2019). The uncertainty in the cloud-screened pyranometer surface forcing estimated at MCOH is ±1.1 W m^−2^ (2.8%).

The CERES science team provides clear sky TOA fluxes. We used the level 3 (1°x 1° gridded) CERES product SYNoptic radiative fluxes and clouds, SYN1deg Ed4A version. ED4 A version is susceptible to least cloud contamination and the TOA clear-sky SW global flux anomaly is within +1 W m^−2^ ^53^. The details of CERES cloud screening are available elsewhere^51,53–55^.

Importantly, we had sufficient cloud-free CERES data samples for significance study. (n_SA_ = 57 for 2020 and n_SA_ = 123 for 2018 & 2019, n_IGP_ = 56 for 2020 and n_IGP_ = 108 for 2018 & 2019 and n_MCOH_ = 34 for 2020 and n_MCOH_ = 71 for 2018 & 2019; n_SA_, n_IGP_, and n_MCOH_ are the number of cloud-free CERES data samples for SA, IGP, and Maldives Climate Observatory Hanimaadhoo). The uncertainty in the TOA clear sky shortwave flux estimated for SA, IGP, and Maldives Climate Observatory Hanimaadhoo are ±0.77 W m^−2^ (1.3%), ±4.5 W m^−2^ (6.1%), ±1.8 W m^−2^ (4.2%), respectively.

The natural variability of aerosols (dust and sea salt) plays a vital role in aerosol radiative forcing over SA. Recent studies^24,39,56^ report the dominance of natural aerosols during the Covid-19 pandemic lockdown and the decline of anthropogenic emissions. Other studies, attribute this reduction in anthropogenic emissions primarily due to a reduction in fossil fuel consumption in the transportation sector^22,36^. Interestingly, no major dust events or amplification of dust transport is reported over SA during MAM-2020. We consider 2020 a normal year for natural aerosols compared to the reference period. However, the surface brightening reported in this study during MAM 2020 is the net effect of demasking of anthropogenic and natural aerosols during the lockdown.

### Modeling of climate-relevant aerosol properties

The radiative effects of aerosols are estimated using the Santa Barbara DISORT Atmospheric Radiative Transfer (SBDART) model^19^, which considers the AOD (from MODIS), SSA (derived from surface measurements and OMI), surface reflectance (from MODIS satellite) asymmetry parameter (modeled), and pressure, temperature, water vapor and ozone profiles from the inbuilt standard tropical atmospheric model^57^. This atmospheric model is well-accepted and is widely used for calculating aerosol radiative forcing estimates over SA^19,58–60^. The asymmetry parameter used in SBDART was modeled using the Optical Properties of Aerosols and Clouds (OPAC) model^61^ following existing studies^62–65^ and more details are included in the supplementary Note 4. The daily mean, clear sky direct aerosol radiative forcing (in the wavelength range 0.3 to 4 µm) for the surface and top of the atmosphere (TOA) were obtained by averaging the instantaneous forcing estimated at an interval of 30 minutes for each day used in this study.

The corresponding equations for direct aerosol radiative forcing (for surface, TOA (F_SUR,TOA_) and Atmosphere (F_ATM_)) and aerosol-induced atmospheric heating rate (∂*T*/∂*t*) are given in Eqs. 1, 2 and 3, respectively^60^. Diurnal and monthly mean estimates of surface and TOA were then calculated. We report monthly mean estimates in this study.

1 $${{\rm{F}}}_{({{\rm{SUR}}},{{\rm{TOA}}})}={{\rm{F}}^{\prime} }_{({{\rm{SUR}}},{{\rm{TOA}}})}-{{\rm{F}}^{{\prime}{\prime}}}_{({{\rm{SUR}}},{{\rm{TOA}}})}$$

2 $${{\rm{F}}}_{{{\rm{ATM}}}}=\,{{\rm{F}}}_{{{\rm{TOA}}}}-{{\rm{F}}}_{{{\rm{SUR}}}}$$

3 $$\frac{\partial T}{\partial t}=\frac{g}{{C}_{P}}\frac{{{\rm{F}}}_{{{\rm{ATM}}}}}{\varDelta p}$$

F′_(SUR,TOA) and_ F″_(SUR,TOA)_ are direct radiative forcing estimates without and with aerosols at the surface and TOA. The parameter g in Eq. 3 stands for acceleration due to gravity and *C*_*p*_ represents the specific heat at constant pressure. Δ*p* is the extent of the vertical atmospheric column influenced by atmospheric heating.

### Hybrid modeling

The most complete set of observations for aerosol radiative forcing calculations used in this study is from Maldives Climate Observatory Hanimaadhoo (MCOH). The surface forcing (SF) and top of the atmosphere forcing (TOAF) calculated at MCOH are from the pyranometer measurements and CERES satellite observations. We performed a hybrid modeling approach combining ground and satellite-based SSA observations. SSA derived from ground-based measurements were used in the 0–2 km layer while SSA from OMI satellite observations were used in the layer 2–100 km. The model-derived monthly averages of TOAF and SF were constrained with the observations at MCOH by altering the vertical distribution of SSA (surface SSA measurements and satellite observations) and the asymmetry parameter. The same model was extended for the regions IGP and SA by constraining the monthly mean TOAF with the corresponding monthly mean CERES observations. The details of model evaluation and related uncertainties are provided in Supplementary Note 4.

### Statistical analysis

The observation-based analysis at MCOH are based on measurements from 2018 to 2020. The consistency of the differences observed between 2020 and the reference period 2018–2019 was affirmed using long-term observations from satellites. For SA and IGP the comparison with satellite-based data was done for two time period 2003–2019 & 2007–2019. These periods were specifically chosen as MODIS AOD and CALIPSO extinction profile data are available only from 2003 and 2007, respectively. We combined all available information for analysis and arrive at statistically significant conclusions that furthermore are consistent between the different data types and periods.

The statistical significance of the changes reported in this study is performed using a Student’s t-test (Welch corrected) at a 5% significance level^66^. Corresponding parameters of each significance test are reported in Supplementary Tables 1–3 and 5.

## Supplementary information

Supplementary Information

## Data Availability

All data needed to evaluate the conclusions in the paper are present in the paper and/or in the Supplementary Information. All satellite data used in the paper are available free to download from Nasa Giovanni website (https://giovanni.gsfc.nasa.gov/giovanni/). Additional data and codes related to this paper may be requested from the corresponding author (Ö.G.). The data will be also available at Bolin Centre Database (https://bolin.su.se/data).
