# Supplementary material for: Aerosol demasking enhances climate warming over South Asia
Source: NPJ Clim Atmos Sci. 2023 May 20;6(1):39. doi: 10.1038/s41612-023-00367-6 (PMC10199435; doi:10.1038/s41612-023-00367-6)
Supplement: Supplementary file 1 — Supplementary Information [file 41612_2023_367_MOESM1_ESM.pdf]

**npj | Climate and Atmospheric Science**

**Supplementary Information for**

# Aerosol demasking enhances climate warming over South Asia

H.R.C.R. Nair[1], Krishnakant Budhavant[2,3], M. R. Manoj[1,3], August Andersson[1], S. K. Satheesh[3,4,5], V. Ramanathan[6] and Örjan Gustafsson[1*]

[1]*Department of Environmental Science and the Bolin Centre for Climate Research, Stockholm University, Stockholm, Sweden*
[2]*Maldives Climate Observatory at Hanimaadhoo, H. Dh. Hanimaadhoo, Republic of the Maldives*
[3]*Divecha Centre for Climate Change, Indian Institute of Science, Bangalore, India*
[4]*Centre for Atmospheric and Oceanic Sciences, Indian Institute of Science, Bangalore, India*
[5]*DST-Centre of Excellence in Climate Change, Indian Institute of Science, Bangalore, India*
[6]*Scripps Institution of Oceanography, University of California San Diego, La Jolla, CA, USA*

## Supplementary Note 1

## Synoptic Meteorology

The spatially averaged daytime near-surface temperature retrieved from ARIS observations showed negligible differences over SA during MAM 2020 (25.6±1.1 °C) compared to MAM 2015 to 2019 (26.3±2.7 °C) (Supplementary Figure 6a). Similarly, the relative humidity (RH) retrieved from Atmospheric Infrared Sounder (ARIS) onboard Aqua satellite showed trivial variations. The spatially averaged RH during MAM 2020 and MAM 2015-2019 was 60.5±3.3 % and 58.1±3.5 % respectively (Supplementary Figure 6b). The average wind speed, temperature and RH measured from 17 different cities in the Indian subcontinent during MAM 2020 is compared with the MAM of the previous year (Figures 2 & 3 under the session Meteorology in[1]) reports that the meteorological influence during the short time span of lockdown in altering the aerosol loading can be ignored[1]. Overall, the synoptic meteorology over SA during MAM 2020 was similar to previous years of MAM[1,2]. The ground observations from the weather station at Maldives Climate Observatory also show negligible variations (Supplementary Table 6 & Supplementary Figure 7). This corroborates that the meteorological influence in confounding the findings of this study can be ignored.

## Supplementary Note 2

## Surface Albedo and Clear Sky Albedo

The surface albedo and clear sky albedo retrieved from the CERES satellite for the period of MAM, 2015 to 2020 are shown in Supplementary Figure 8. The mean of surface albedo and clear sky albedo during MAM 2015 to 2019 (MAM 2020) is 0.1 ± 0.09 (0.1 ± 0.04) and 0.14 ± 0.09 (0. 14 ± 0.03) respectively. Variations in the flux at the top of the atmosphere (TOA) following changes in the aerosol optical depth (AOD) can be partly

offset by a reduction in the amount of black carbon (BC)[3] which in turn restricts the magnitude of changes in the albedo.

## Supplementary Note 3

### Pyranometer Data Correction

The incoming surface-reaching solar radiation measurements in the wavelength range of 0.28 to 2.8 µm were measured at MCOH using a pyranometer (CMP21, Kipp and Zonen). The instrument is capable of accurately measuring the rapidly changing irradiance with a shorter time constant (<5 s for 95% response). The instrument's absolute accuracy (zero offsets) is ~ ±7 $Wm^{-2}$. Our observations show an absolute accuracy of ~ 0.5±3 W $m^{-2}$. More technical specifications of the instrument can be found elsewhere[4].

The daily raw data from the pyranometer has a temporal resolution of 3 mins. The daily raw data of an ideal clear sky day (Supplementary Figure 9a) was used to estimate the threshold for cloud screening. The difference in insolation between adjacent points computed using this clear sky day data is shown in Supplementary Figure 9b. It was found that the magnitude of this difference is time-dependent and with maximum variations (~20 W $m^{-2}$) when the sun is close to the horizon and minimum variations (~0.5 W $m^{-2}$) when the sun is near the zenith position. In the presence of clouds, the magnitudes of these variations would be much higher (> 30 W $m^{-2}$). A sample measurement with intermittent cloud presence is shown in Supplementary Figure 8c. The difference between adjacent points is shown in Supplementary Figure 9d. The measurement points with a difference greater than 20 W $m^{-2}$ in comparison to the adjacent point can be detected as clouds and removed. This is shown in Supplementary Figure 9e. The cloud-removed data points were smoothed hourly (20 points) (Supplementary Figure 9f) and used for further analysis. A similar approach was used by[5] for cloud screening pyrheliometer data. The radiation measurements corresponding to values of the cosine of solar zenith angle below 0.2 were discarded[6]. The days with less than 60 % cloud-free points (with magnitude above 400 W $m^{-2}$) during local time 08:00 hrs to 16:00 hrs[5] were not used for analysis.

## Supplementary Note 4

### Hybrid Model Evaluation

In the first stage of model optimization, we performed a model simulation for selected clear sky days of the study period by varying the vertical distribution of single scattering albedo (SSA) and the asymmetry parameter. We individually estimated top of the atmosphere forcing (TOAF) and surface forcing (SF) for the selected dates for different combinations of asymmetry parameter and SSA. The aerosol asymmetry parameter was modeled using the Optical Properties of Aerosols and Clouds (OPAC) model[7]. OPAC model estimates aerosol optical properties based on the chemistry and particle number concentrations[7]. OPAC provides options to vary the relative humidity conditions based on the location of the study[7]. Component-specific aerosol number concentrations of organic carbon, mineral dust, water-soluble ions, black carbon and sea salt were used as input to the OPAC model[7,8]. These component-specific inputs are not a complete mass closure of water-soluble and water-insoluble materials[8]. This is compensated by tuning the number concentrations to obtain OPAC outputs to match with the observations. The number concentrations were tuned iteratively until the OPAC-modeled AOD and SSA constrain with less than ~5% deviation with surface observations[8,9]. This is a widely accepted method and is used by many recent studies[8–11]. For constraining the SBDART model, simulations were performed using surface-based SSA for the layer close to the ground. The selected altitudes ranged from 0.5 km to 3 km with a step interval of 0.5 km. For the layer above this (top of bottom layer to 100 km) SSA from satellite observations were used. The averages of each combination were compared with CERES and pyranometer observations and the best match was obtained for altitudes up to 2 km with surface SSA and from 2 km to 100 km using satellite-derived SSA (Supplementary Figure 4, Supplementary Table 4). To verify the robustness of the model, we ran simulations for the rest of the days and confirmed that the model output matches with the CERES and pyranometer values. The overall uncertainty in model computed SF and TOAF compared to observations are estimated to be ~ 1% and 12. 6 % respectively. The same model was extended for studying the forcing variations over Indo Gangetic Plain and South Asia.

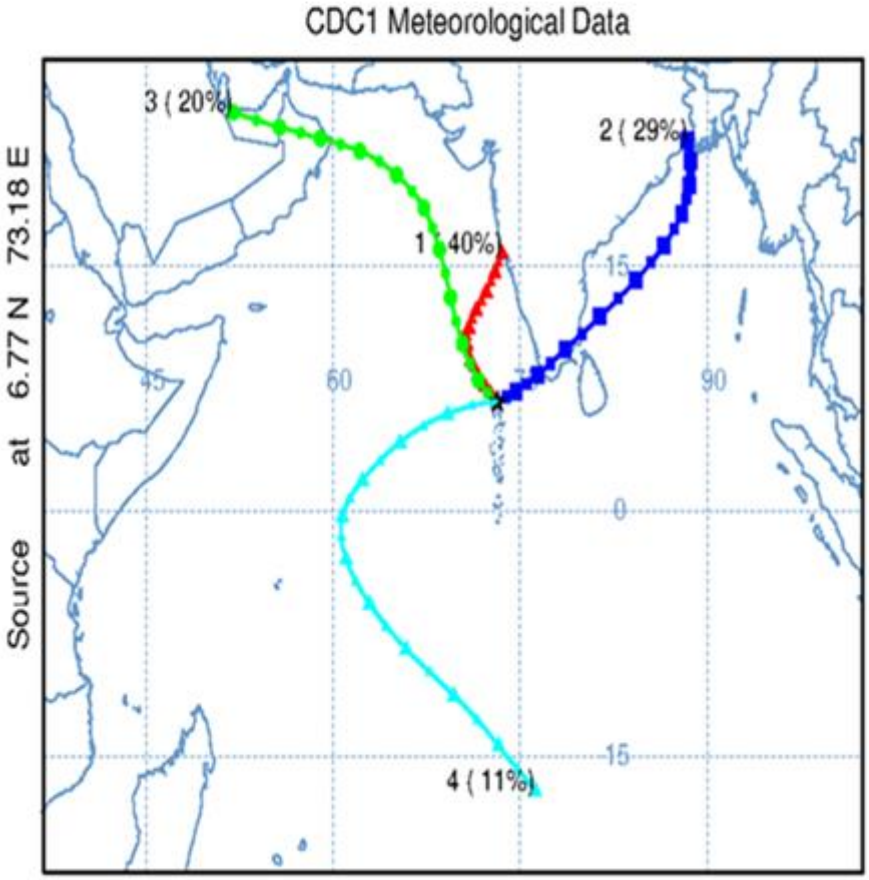


**Supplementary Figure 1. Air mass trajectories.** The mean trajectories of the air masses passing over Hanimaadhoo (MCOH) at an altitude of 100 m during the period December to May (2017-20). A total of 2535 trajectories were averaged and four different geographical locations contributing significantly to MCOH were identified. These locations and the trajectories of the air masses arriving from these locations are represented by the four colored lines. The number of clusters arriving from Indo Gangetic Plain/Bay of Bengal (blue line), South India/Western coast of India (red line), Arabian Peninsula (green line) and Indian Ocean (cyan line) are respectively 728, 1024, 504 and 279.

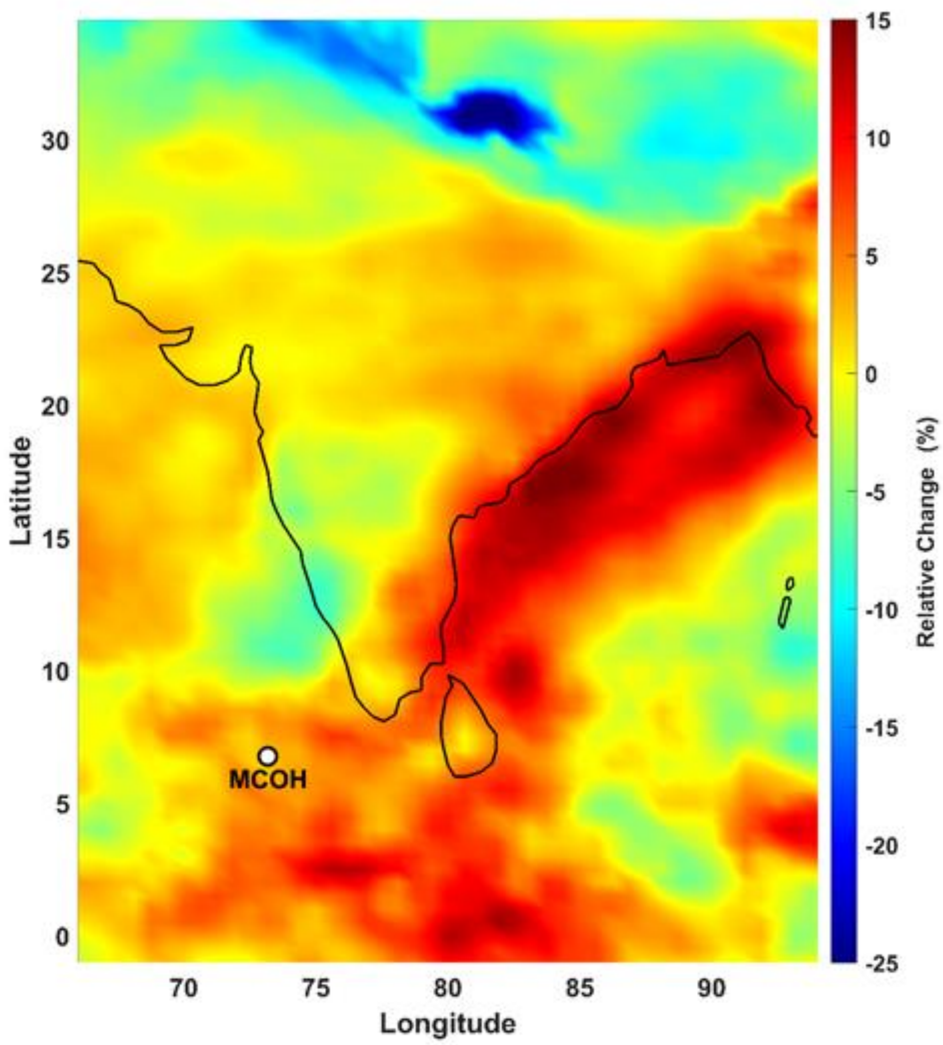


**Supplementary Figure 2. Relative change in incoming surface-reaching shortwave solar radiation.** Data was obtained from the MERRA reanalysis. The relative change shown here (in percentage) represents the change in the values during April-May 2020 with respect to the mean value during the period April-May 2015 to 2019.

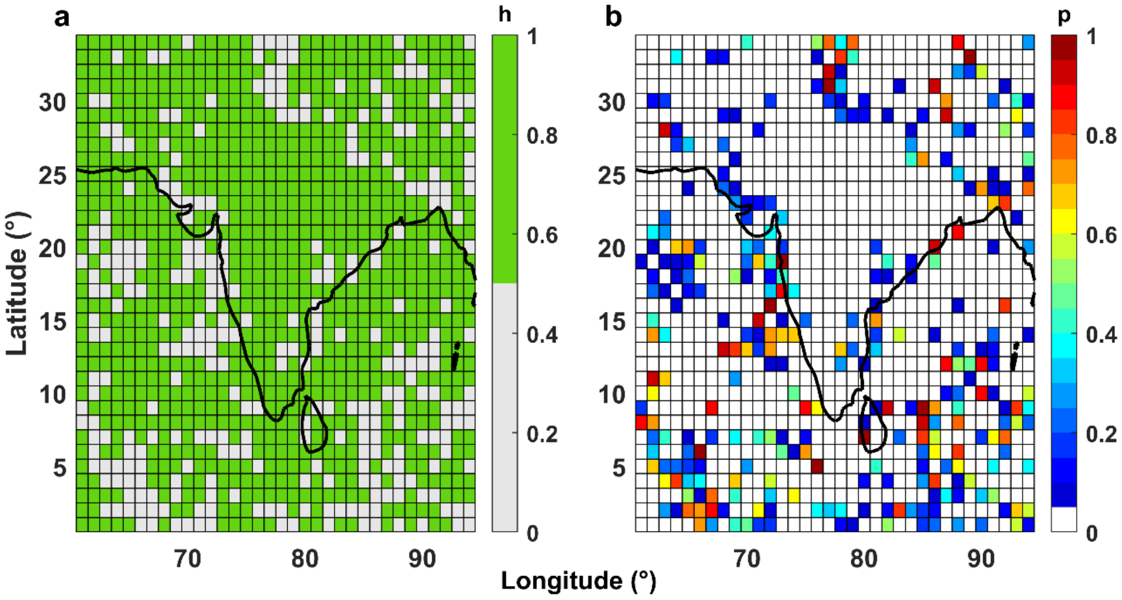


**Supplementary Figure 3. Statistical significance of CERES TOA flux over South Asia.** A grid to grid t-test was performed using data from each 1°x1° grid using periods 2007-2019 and 2020. **(a)** shows the h value of the null hypothesis that the means of the distribution are the same at 95% confidence level. The green color represents h=1 which shows that there is a significant change in the majority of the grids during the year 2020. **(b)** The corresponding p values.

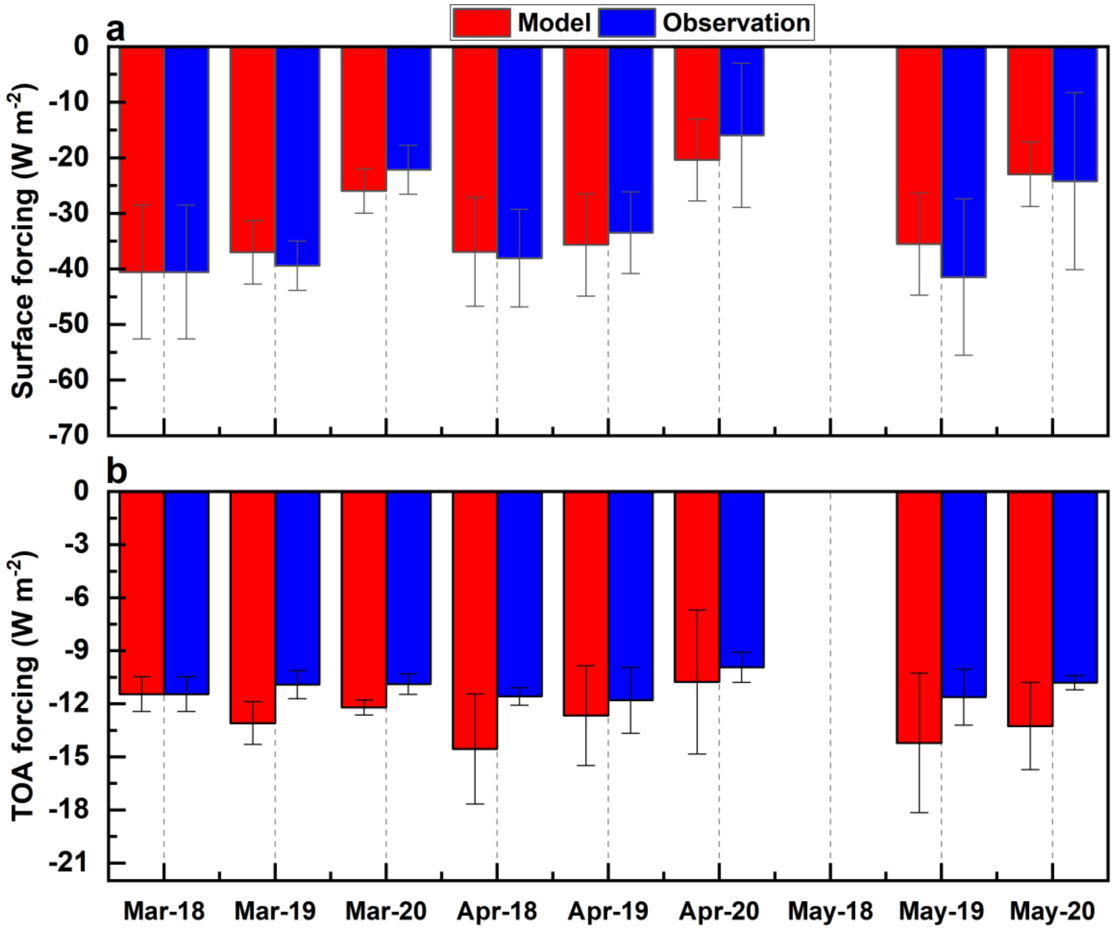


**Supplementary Figure 4. Aerosol radiative forcing.** Monthly average aerosol radiative forcing over Maldives Climate Observatory-Hanimaadhoo (MCOH) from March to May (2018-2020) **(a)** estimated using ground-based pyranometer measurements (at the surface) and CERES satellite observations (at the top of the atmosphere) and **(b)** calculated using SBDART. Vertical black color error bars indicate the standard deviations.

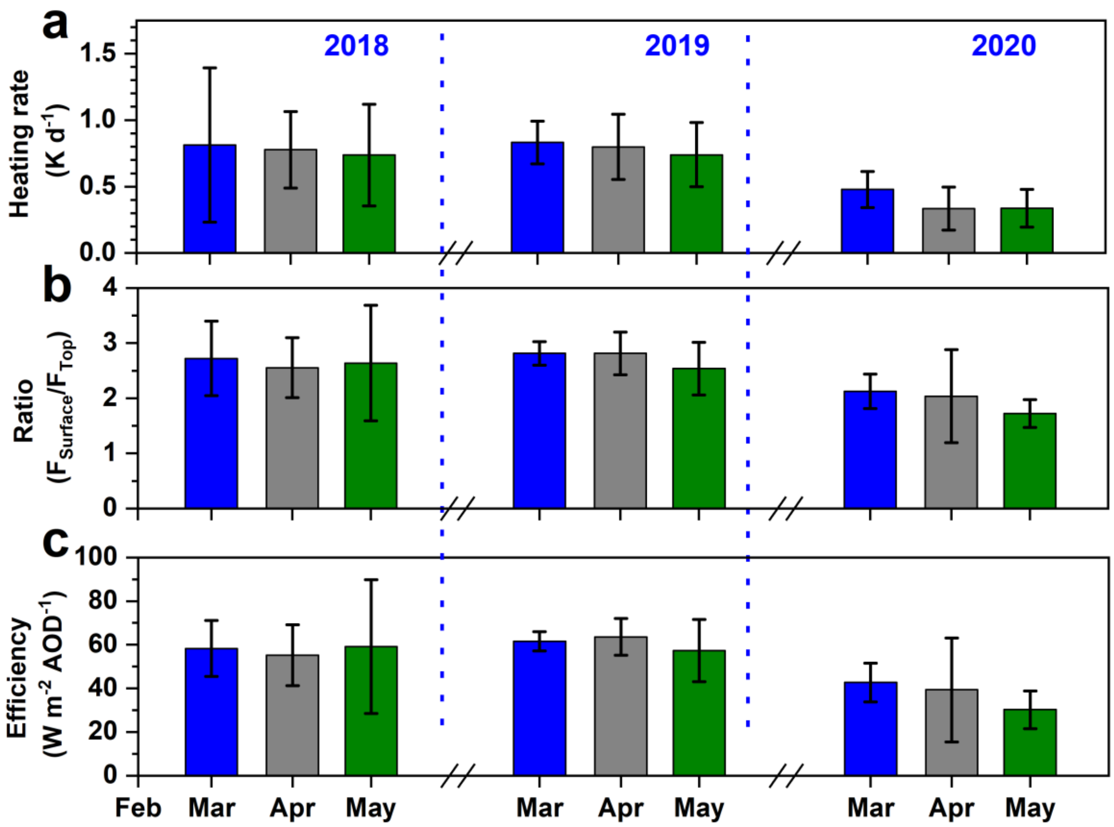


**Supplementary Figure 5. Radiative effects of demasking.** The monthly average **(a)** atmospheric heating rate, **(b)** the ratio of surface forcing to the top of the atmosphere, and **(c)** atmospheric radiative forcing efficiency over Maldives Climate Observatory-Hanimaadhoo (MCOH) during March-May (2018-2020). Vertical black color error bars indicate the standard deviation.

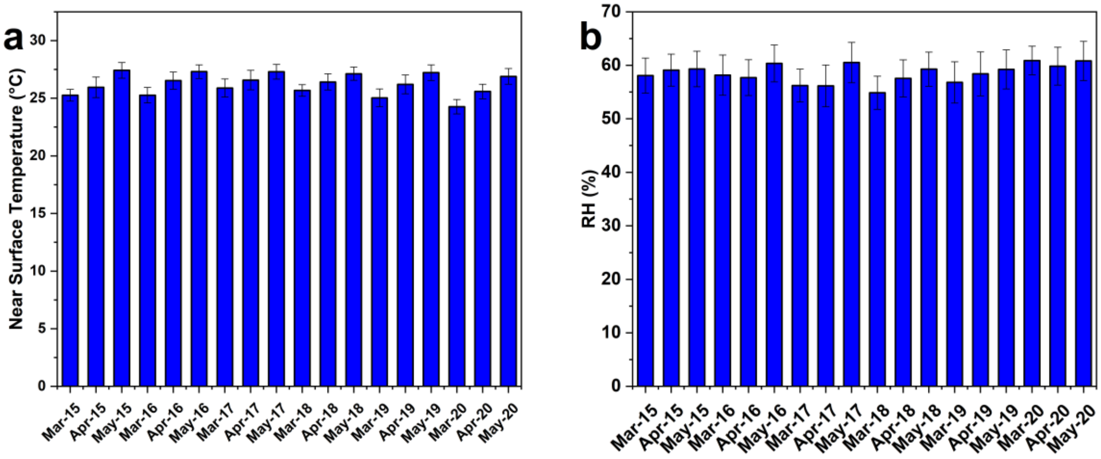


**Supplementary Figure 6. Changes in near-surface temperature and relative humidity.** Monthly mean variations of **(a)** near-surface temperature and **(b)** monthly mean relative humidity over South Asia during the period 24th March to 31st May (2015 to 2020) were retrieved using Atmospheric Infrared Sounder (ARIS) onboard the Aqua satellite (AIRS3STD, version 7.0, 1°× 1° resolution). Vertical black color error bars indicate the standard deviations.

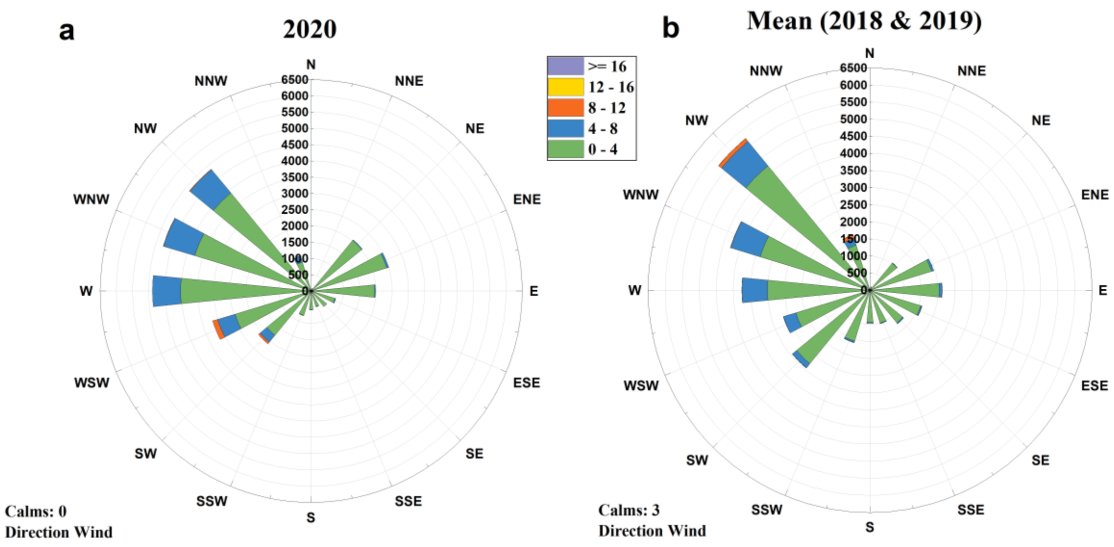


**Supplementary Figure 7. Changes in surface wind.** Mean wind speed and wind direction obtained from the weather station at Maldives Climate Observatory (MCOH). **(a)** Mean wind speed and wind direction during 24 March to 31 May-2020, **(b)** Mean wind speed and wind direction during 24 March to 31 May, 2015 to 2019.

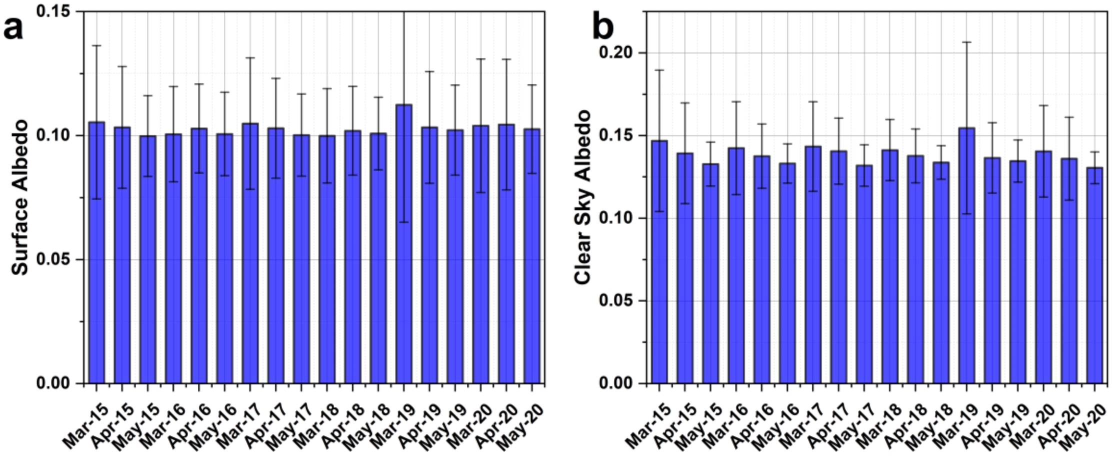


**Supplementary Figure 8. Changes in Albedo.** Monthly mean variations of **(a)** Surface albedo and **(b)** Clear sky albedo over South Asia during the period 24th March to 31st May (2015 to 2020) were retrieved using Clouds and the Earth's Radiant Energy System (CERES) instruments on board the Terra and Aqua satellites (Terra + Aqua Edition 4.1 SYN1deg dataset, 1°× 1° resolution, Version 4.1). Vertical black color error bars indicate the standard deviations.

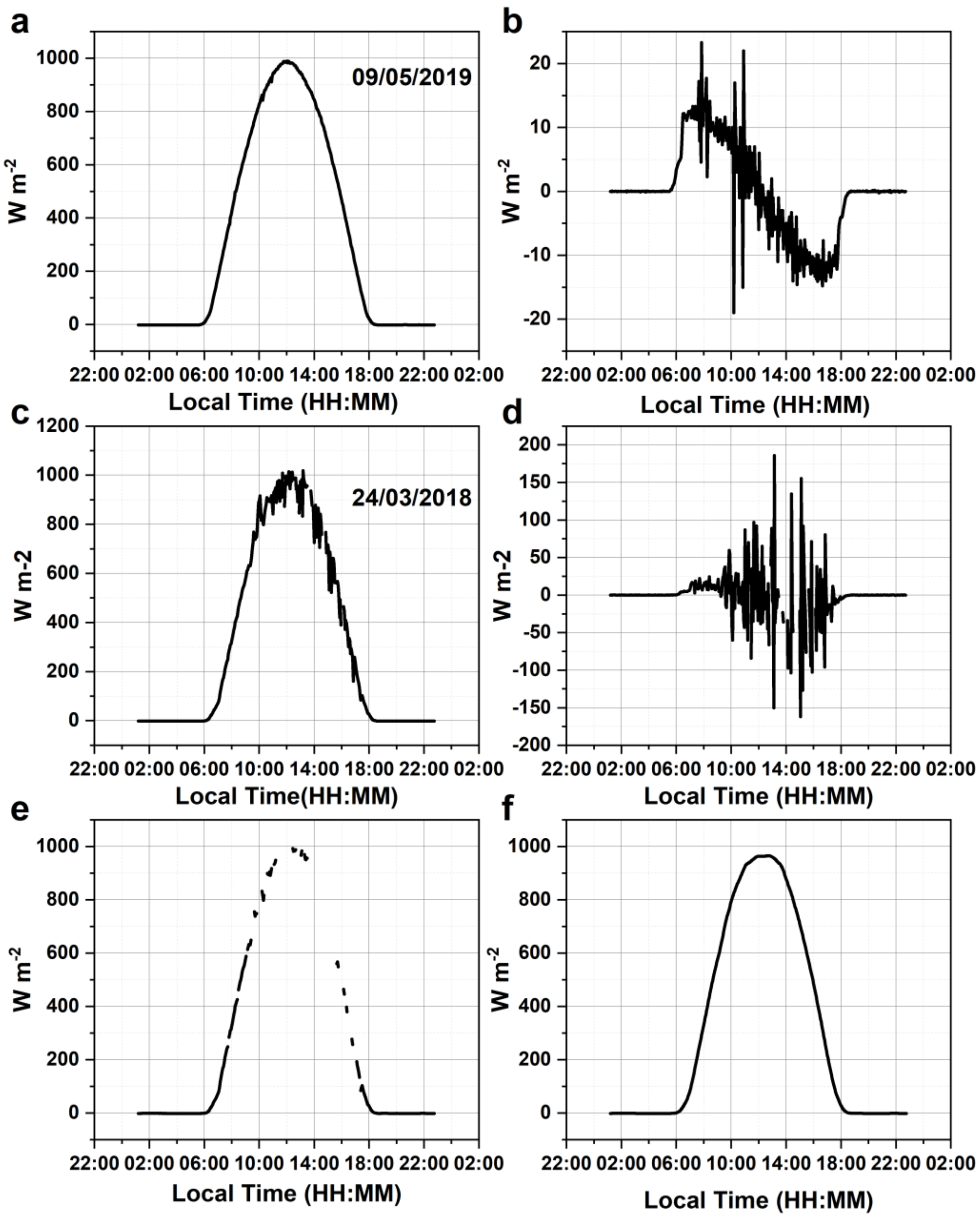


**Supplementary Figure 9. Pyranometer Data Correction.** Incoming shortwave radiation was measured using a pyranometer at MCOH (3 min resolution). **(a)** Clear sky day measurements, **(b)** Difference in insolation between adjacent data points of panel A, **(c)** Intermittent cloudy day measurements, **(d)** Difference in insolation between adjacent data points of panel **c**, **(e)** Cloud screened data points of the intermittent cloudy day (panel **c**) and **(f)** Final cloud corrected data corresponding to panel **c**.

**Supplementary Table 1. Satellite-derived aerosol parameters and radiative forcing over the Indo-Gangetic Plain and South Asia.** Retrieved during the period 24 March to 31 May 2020 and the corresponding periods of 2018 and 2019.

| | **Indo-Gangetic Plain** | | | **South Asia** | | |
|---|---|---|---|---|---|---|
| Period | Aerosol optical depth | TOA Flux | TOA Forcing | Aerosol optical depth | TOA Flux | TOA Forcing |
| | MODIS (550 nm) | CERES (W $m^{-2}$) | CERES (W $m^{-2}$) | MODIS (550 nm) | CERES (W $m^{-2}$) | CERES (W $m^{-2}$) |
| 2018-19 | 0.57 ± 0.1 | 74.7 ± 3.8 | -14.3 ± 1.7 | 0.41 ± 0.1 | 58.5 ± 0.7 | -10.8 ± 0.7 |
| 2020 | 0.47 ± 0.1 | 72.9 ± 2.6 | -12 ± 1.3 | 0.34 ± 0.05 | 57.3± 0.4 | -9.4 ± 0.5 |
| *t | 4.9 | 3.3 | -9.7 | 8.4 | 15.5 | -14.5 |
| *p | $3.6x10^{-6}$ | 0.001 | $2.5x10^{-17}$ | 1.8x10-14 | 8.3x10-34 | 6.7x10-30 |

* The statistical significance of the changes observed during the period 24 March to 31 May 2020 is represented using a Student's t-test (Welch corrected) at a 5% significance level.

**Supplementary Table 2.** The satellite-derived aerosol parameters over Indo-Gangetic Plain, South Asia, and Maldives Climate Observatory Hanimaadhoo. Retrieved during 24 March to 31 May of 2003-2019 and 2007-2019.

| | **Indo-Gangetic Plain** | | **South Asia** | | **Maldives Climate Observatory Hanimaadhoo** | |
|---|---|---|---|---|---|---|
| | Aerosol optical depth | TOA Flux | Aerosol optical depth | TOA Flux | Aerosol optical depth | TOA Flux |
| | MODIS (550 nm) | CERES (W $m^{-2}$) | MODIS (550 nm) | CERES (W $m^{-2}$) | MODIS (550 nm) | CERES (W $m^{-2}$) |
| 2003-2019 | 0.51 ± 0.1 | 77.3 ± 4.2 | 0.37 ± 0.1 | 59.9 ± 1.2 | 0.34 ± 0.1 | 41.6 ± 1.9 |
| *t | 2.9 | 8 | 5.1 | 9.8 | 3.4 | 4.3 |
| *p | 0.005 | 1.001 x$10^{-11}$ | 2.7 x $10^{-6}$ | 3.1 x $10^{-15}$ | 0.001 | 7.9 x $10^{-5}$ |
| 2007-2019 | 0.52 ± 0.1 | 76.9 ± 4 | 0.38 ± 0.06 | 58.8 ± 1.3 | 0.36 ± 0.1 | 41.8 ± 2 |
| t | 3.6 | 6.9 | 7.1 | 9.5 | 2.81 | 4.9 |
| p | 4.9 x $10^{-4}$ | 1.27 x $10^{-9}$ | 2.3 x $10^{-10}$ | 6.9 x $10^{-15}$ | 0.007 | 7.1 x $10^{-6}$ |

* The statistical significance of the changes observed during the period 24 March to 31 May 2020 is represented using a Student's t-test (Welch corrected) at a 5% significance level.

**Supplementary Table 3. Aerosol parameters measured/satellite retrieved/calculated over MCOH.** Corresponding to 24 March to 31 May 2020 and the corresponding periods of 2018 and 2019.

| | **Columnar Observations** | | **Surface Measurements** | | |
|---|---|---|---|---|---|
| **Period** | **Aerosol optical depth** | **Incoming solar radiation** | **Black carbon** | **Particle number** | **Single Scattering Albedo** |
| | **AERONET (Level-2.0)** | **Pyranometer ($W\ m^{-2}$)** | **Aethalometer ($ng\ m^{-3}$)** | **CPC ($\#\ cm^{-3}$)** | |
| **2018-19** | 0.42 ± 0.10 | 278.3 ± 8.7 | 1160 ±471 | 1590 ± 402 | 0.86 ± 0.04 |
| **2020** | 0.28 ± 0.08 | 298.3 ± 9.1 | 470 ± 134 | 832 ± 323 | 0.95 ± 0.03 |
| ***t** | 4.6 | -5.9 | 6.6 | 8.7 | -8.17 |
| ***p** | $1.6x10^{-5}$ | $2.1x10^{-7}$ | $2.3x10^{-10}$ | $9.5x10^{-16}$ | $1.1x10^{-11}$ |

| | **Radiative Forcing** | | **Satellite Measurements** | |
|---|---|---|---|---|
| **Period** | **TOA Forcing** | **Surface Forcing** | **Aerosol optical depth** | **TOA Flux** |
| | **CERES ($W\ m^{-2}$)** | **Pyranometer ($W\ m^{-2}$)** | **MODIS (550 nm)** | **CERES ($W\ m^{-2}$)** |
| **2018-19** | -11.4 ± 1.6 | -37.9 ± 10.8 | 0.38 ± 0.1 | 42.06 ± 1.62 |
| **2020** | -10.4 ± 0.8 | -19.8 ± 13.9 | 0.29 ± 0.09 | 41.02 ± 0.78 |
| **t** | -4.3 | -6.7 | 4.6 | 4.4 |
| **p** | $3.9x10^{-5}$ | $1.4x10^{-8}$ | $2.2x10^{-5}$ | $2.1x10^{-5}$ |

* The statistical significance of these changes with respect to the COVID period is represented using a student's t-test (Welch corrected) at a 5% significance level.

**Supplementary Table 4. Statistical details of aerosol radiative forcing at MCOH.** Data obtained from model and measurements during the period 24 March to 31 May.

| Year | *SBDART Model Output | Pyranometer Ground Observation | *SBDART Model Output | CERES Satellite Observation |
|---|---|---|---|---|
| | Surface Forcing ($W\ m^{-2}$) | Surface Forcing ($W\ m^{-2}$) | TOA Forcing ($W\ m^{-2}$) | TOA Forcing ($W\ m^{-2}$) |
| **2018-2019** | -36.1 ± 10.1 | -37.9 ± 10.8 | -13.6 ± 3.4 | -11.4 ± 1.6 |
| **2020** | -22 ± 6.6 | -19.8 ± 13.9 | -11.9 ± 3.4 | -10.4 ± 0.8 |

*The radiative effects of aerosols are estimated using the Santa Barbara DISORT Atmospheric Radiative Transfer (SBDART) model.

**Supplementary Table 5. Aerosol radiative forcing modeled over Maldives Climate Observatory-Hanimaadhoo, Indo-Gangetic Plain and South Asia.** Corresponding to 24 March to 31 May 2020 and the corresponding periods of 2018 and 2019.

| Period | Top of the Atmosphere | Surface | Atmosphere | Atmospheric Forcing Efficiency (W m$^{-2}$ AOD$^{-1}$) | $\frac{F_{SURF}}{F_{TOP}}$ | Atmospheric heating rate (K d$^{-1}$) |
|---|---|---|---|---|---|---|
| | Forcing (W m$^{-2}$) | | | | | |
| **Maldives Climate Obserevatory-Hanimaadhoo** | | | | | | |
| **2018-19** | -13.6 ± 3.4 | -36.1 ± 10.1 | 22.5 ± 8 | 59 ± 12.9 | 2.7 ± 0.5 | 0.8 ± 0.3 |
| **2020** | -11.9 ± 3.4 | -22 ± 6.6 | 10.1 ± 4.4 | 36.3 ± 18.3 | 1.9 ± 0.6 | 0.4 ± 0.3 |
| ***t** | -2.4 | -8.5 | 10.1 | 6.5 | 5.9 | 10.1 |
| ***p** | 0.02 | $2.9 \times 10^{-13}$ | $4.9 \times 10^{-17}$ | $3.3 \times 10^{-8}$ | $2.4 \times 10^{-7}$ | $4.9 \times 10^{-17}$ |
| **Indo-Gangetic Plain** | | | | | | |
| **2018-19** | -15.3 ± 3.8 | -46.3 ± 8.7 | 31 ± 6.9 | 54.9 ± 8.9 | 3.1 ± 0.5 | 1.1 ± 0.2 |
| **2020** | -13.1 ± 3.5 | -30.5 ± 8.5 | 17.3 ± 7.3 | 36.7 ± 11.7 | 2.4 ± 0.6 | 0.6 ± 0.2 |
| **t** | -3.6 | -11.2 | 11.6 | 10.2 | 7.5 | 11.6 |
| **p** | $4.7 \times 10^{-4}$ | $3.8 \times 10^{-20}$ | $1.4 \times 10^{-20}$ | $1.5 \times 10^{-16}$ | $3.8 \times 10^{-11}$ | $1.4 \times 10^{-20}$ |
| **South Asia** | | | | | | |
| **2018-19** | -12.3 ± 2.6 | -34.7 ± 5.5 | 22.4 ± 4.7 | 55.1 ± 9.9 | 2.9 ± 0.5 | 0.8 ± 0.2 |
| **2020** | -10.9 ± 1.9 | -23 ± 4.2 | 12.1 ± 4.2 | 36.1 ± 11.9 | 2.2 ± 0.5 | 0.4 ± 0.2 |
| **t** | -4.2 | -15.6 | 14.6 | 10.5 | 9.3 | 14.6 |
| **p** | $4.3 \times 10^{-5}$ | $2.2 \times 10^{-32}$ | $3.3 \times 10^{-28}$ | $1.5 \times 10^{-17}$ | $1.8 \times 10^{-15}$ | $3.3 \times 10^{-28}$ |

* The statistical significance of the changes observed during the period 24 March to 31 May 2020 is represented using a Student's t-test (Welch corrected) at a 5% significance level.

**Supplementary Table 6. Synoptic Meteorology.** Statistical details of different parameters were measured using the weather station at the Maldives Climate Observatory- Hanimaadhoo (MCOH)

| **Period** | **Temperature (°C)** | **Pressure (hPa)** | **Relative Humidity (%)** | **Wind Speed (m $s^{-1}$)** |
|---|---|---|---|---|
| **2018-19** | 29.4 ±1.4 | 1007.5 ±1.8 | 74.1 ±6.4 | 2.4 ±1.5 |
| **2020** | 29.5 ±1.3 | 1007.8 ±1.6 | 72.7 ±6.4 | 2.5 ±1.5 |

## Supplementary References


1. Navinya, C., Patidar, G. & Phuleria, H. C. Examining Effects of the COVID-19 National Lockdown on Ambient Air Quality across Urban India. *Aerosol Air Qual. Res.* **20**, 1759–1771 (2020).
2. Sharma, S. *et al.* Effect of restricted emissions during COVID-19 on air quality in India. *Sci. Total Environ.* **728**, 138878 (2020).
3. Rutan, D. *et al.* Development and assessment of broadband surface albedo from Clouds and the Earth's Radiant Energy System Clouds and Radiation Swath data product. *J. Geophys. Res. Atmos.* **114**, 8125 (2009).
4. Manual of Kipp & Zonen's Pyranometer range - Kipp & Zonen. https://www.kippzonen.com/Download/72/Manual-Pyranometers-CMP-series-English?ShowInfo=true.
5. Vuilleumier, L. *et al.* Accuracy of ground surface broadband shortwave radiation monitoring. *J. Geophys. Res. Atmos.* **119**, 13,838-13,860 (2014).
6. Cess, R. D., Qian, T. & Sun, M. Consistency tests applied to the measurement of total, direct, and diffuse shortwave radiation at the surface. *J. Geophys. Res. Atmos.* **105**, 24881–24887 (2000).
7. Hess, M., Koepke, P. & Schult, I. Optical Properties of Aerosols and Clouds: The Software Package OPAC. *Bull Am. Meteorol. Soc.* **79**, 831–844 (1998).
8. Wang, Q. *et al.* Measurement report: Source and mixing state of black carbon aerosol in the North China Plain: Implications for radiative effect. *Atmos. Chem. Phys.* **20**, 15427–15442 (2020).
9. Liu, H. *et al.* Changes in Source-Specific Black Carbon Aerosol and the Induced Radiative Effects Due to the COVID-19 Lockdown. *Geophys. Res. Lett.* **48**, e2021GL092987 (2021).
10. Bibi, S., Alam, K., Chishtie, F., Bibi, H. & Rahman, S. Observations of black carbon aerosols characteristics over an urban environment: Radiative forcing and related implications. *Sci. Total Environ.* **603–604**, 319–329 (2017).
11. Pathak, B., Kalita, G., Bhuyan, K., Bhuyan, P. K. & Moorthy, K. K. Aerosol temporal characteristics and its impact on shortwave radiative forcing at a location in the northeast of India. *J. Geophys. Res. Atmos.* **115**, 19204 (2010).
